# Deconvoluting AMP-activated protein kinase (AMPK) adenine nucleotide binding and sensing

**DOI:** 10.1074/jbc.M117.793018

**Published:** 2017-06-14

**Authors:** Xin Gu, Yan Yan, Scott J. Novick, Amanda Kovach, Devrishi Goswami, Jiyuan Ke, M. H. Eileen Tan, Lili Wang, Xiaodan Li, Parker W. de Waal, Martin R. Webb, Patrick R. Griffin, H. Eric Xu, Karsten Melcher

**Affiliations:** From the ‡ Laboratories of Structural Sciences and Structural Biology and Biochemistry, Center for Cancer and Cell Biology, Van Andel Research Institute, Grand Rapids, Michigan 49503,; the § VARI-SIMM Center, Center for Structure and Function of Drug Targets, The CAS Key Laboratory of Receptor Research, Shanghai Institute of Materia Medica, Chinese Academy of Sciences (CAS), Shanghai 201203, China,; the ¶ Department of Molecular Medicine, Translational Research Institute, The Scripps Research Institute, Jupiter, Florida 33458, and; ‖ The Francis Crick Institute, 1 Midland Road, London NW1 1AT, United Kingdom

**Keywords:** AMP, AMP-activated kinase (AMPK), ATP, energy metabolism, protein kinase, CBS, HDX-MS, NADPH

## Abstract

AMP-activated protein kinase (AMPK) is a central cellular energy sensor that adapts metabolism and growth to the energy state of the cell. AMPK senses the ratio of adenine nucleotides (adenylate energy charge) by competitive binding of AMP, ADP, and ATP to three sites (CBS1, CBS3, and CBS4) in its γ-subunit. Because these three binding sites are functionally interconnected, it remains unclear how nucleotides bind to individual sites, which nucleotides occupy each site under physiological conditions, and how binding to one site affects binding to the other sites. Here, we comprehensively analyze nucleotide binding to wild-type and mutant AMPK protein complexes by quantitative competition assays and by hydrogen-deuterium exchange MS. We also demonstrate that NADPH, in addition to the known AMPK ligand NADH, directly and competitively binds AMPK at the AMP-sensing CBS3 site. Our findings reveal how AMP binding to one site affects the conformation and adenine nucleotide binding at the other two sites and establish CBS3, and not CBS1, as the high affinity exchangeable AMP/ADP/ATP-binding site. We further show that AMP binding at CBS4 increases AMP binding at CBS3 by 2 orders of magnitude and reverses the AMP/ATP preference of CBS3. Together, these results illustrate how the three CBS sites collaborate to enable highly sensitive detection of cellular energy states to maintain the tight ATP homeostastis required for cellular metabolism.

## Introduction

ATP homeostasis is critically important for cellular functions and is maintained at a tight concentration range by the regulatory protein kinase AMP-activated protein kinase (AMPK), ^2^ a therapeutic target for metabolic diseases and cancer (1–7). Because of this tight range, AMPK senses the energy state of the cell largely as ratio of AMP to ATP, which changes much more dramatically than ATP levels under energy stress, whereas ADP levels change relatively more modestly. High levels of AMP activate AMPK by three different mechanisms: direct allosteric activation of its kinase activity (8, 9), increasing activation loop phosphorylation by upstream kinase (10, 11), and protection of the phosphorylated activation loop against dephosphorylation (12, 13). High levels of ADP also protect against activation loop dephosphorylation (although 5–10 times less potently than AMP for the γ1 and γ3 isoforms) and do not stimulate direct kinase activation as AMP (8, 14–17).

AMPK consists of three subunits, a kinase domain-containing α-subunit, a carbohydrate-binding module (CBM)-containing β-subunit, and an adenine nucleotide-binding γ-subunit. The γ-subunit contains four cystathionine β-synthetase (CBS) motifs, which function in pairs known as Bateman domains as universal adenine nucleotide-binding sites (18). CBS1 and CBS2 form Bateman domain 1, whereas CBS3 and CBS4 form Bateman domain 2 (note that CBS1, -2, -3, and -4 were previously (19) known as sites 2, 4, 1, and 3). Crystal structures and binding assays have established that CBS2, in which the conserved, ribose-binding aspartate is mutated to arginine, fails to bind adenine nucleotides (15, 19–21). On the other hand, AMP is tightly bound to CBS4 (19, 20) as wild-type AMPK and cbs1 and cbs3 mutant AMPK co-purify with an equimolar amount of AMP, whereas a cbs4 mutant only purifies with trace amounts of nucleotides (20). Therefore CBS4 likely does not exchange AMP against ATP in the presence of physiological AMP levels (19). However, under artificial conditions, *i.e.* the complete absence of AMP, prebound AMP at CBS4 can very slowly exchange against ATP in solution (∼40% exchange after removal of free nucleotides, followed by 6 h incubation with 5 mm ATP) (20). The remaining two CBS sites (CBS1 and CBS3) are thought to be exchangeable as biochemical binding and competition experiments confirmed the existence of two readily exchangeable adenine nucleotide (AXP)-binding sites in purified recombinant AMPK (15, 18, 19, 22). Mutations in key AXP-binding residues in the γ2- and γ3-AMPK isoforms increase basal AMPK activity and are causally associated with glycogen storage diseases and Wolf-Parkinson-White cardiomyopathy (23–30).

AMP-occupied CBS3 is directly bound by a loop (αRIM) adjacent to the autoinhibitory domain of the α-subunit (15, 31), suggesting that the αRIM senses AMP at CBS3. Consistently, mutating key αRIM residues compromises AMPK activation (31). Moreover, AMP increases and ATP decreases the interaction between the isolated αRIM loop and core-AMPK (γ-subunit plus scaffolding C termini of the α- and β-subunit), and AMP stimulation of the interaction requires the intact αRIM (32). In addition to mutations in CBS3, also mutations in CBS4, which does not interact with the αRIM or other parts of the α- or β-subunits, abolish allosteric kinase activation by AMP (20), yet the molecular basis for the CBS4 requirement has remained elusive. When ATP was soaked into crystals of AMP-bound core-AMPK, ATP occupied CBS1 and CBS3, whereas CBS4 remained AMP bound (19, 20). In contrast, when AMP-depleted core-AMPK was co-crystallized with ATP, ATP bound to CBS1 and CBS4, CBS3 was unoccupied, and the γ-subunit underwent a noticeable conformational change (20).

One of the two exchangeable AXP-binding sites binds both AMP and Mg^2+^-free ATP with micromolar affinity (“high affinity site”), whereas the other site binds AXP much more weakly (hundreds of micromolar (15, 19, 22)), and likely with positive cooperativity (18). Recent surface plasmon resonance (Biacore) data have revealed that ATP binds AMPK in the presence of Mg^2+^, which shields charge interactions of ATP phosphates with CBS residues, with single site kinetics and a binding constant in the 100 μm range (22). This low affinity of Mg^2+^-ATP would be consistent with preferential AMP-binding at the high affinity binding site to allow AMP exchange at nucleotide concentrations that reflect cellular conditions of energy excess and energy stress (14). Currently, it is poorly understood which affinity state maps to which CBS site, how nucleotide binding at one site affects binding at the other site(s), and what adenine nucleotide binds to each of the three functional CBS sites at physiological concentrations of ATP, ADP, and AMP. Focusing on AMP and ATP, we combined fluorescence binding and competition experiments of wild-type and mutant AMPK with hydrogen deuterium exchange mass spectrometry (HDX-MS). These experiments allowed us to (i) identify CBS3 as a high affinity exchangeable AXP-binding site as well as binding site for NADPH, (ii) map the CBS conformational connectivity and demonstrate that preferential AMP-binding at CBS3 requires AMP-bound CBS4, and (iii) suggest that under physiological conditions only CBS3 significantly exchanges between AMP and ATP.

## Results

### Isolated CBS motifs bind AXP weakly and with altered AMP/ATP specificity

**Figure 1.**
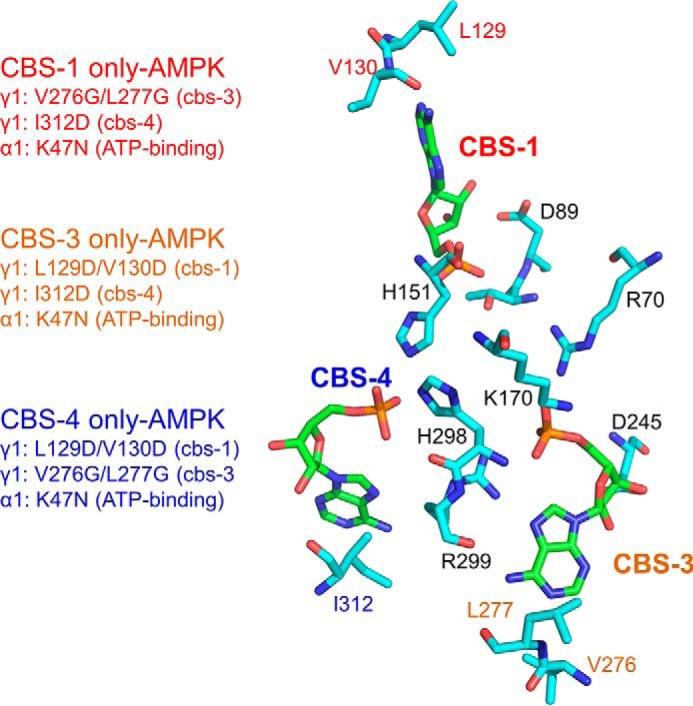
**Binding of AMP to the γ1 subunit.** Stick presentation of the three AMP molecules (*green carbon atoms*) and key AMP-binding residues of AMPK (*cyan carbon atoms*). Oxygen is colored in *red*, nitrogen in *blue*, and phosphorus in *orange*. The phosphate groups of the 3 AMP molecules are coordinately bound by a set of charged amino acids, whereas the ribose and adenine rings face away from each other and are bound by CBS-specific residues. Mutations of the indicated adenine-binding residues therefore selectively block AMP binding to individual CBS sites. Note that the adenine-binding mutations at CBS3 and CBS4 are each sufficient, even at the high concentration of adenine nucleotides in cells, to completely abolish AMPK activation by AMP without affecting AMPK catalytic activity in the absence of AMP (18).

**Figure 2.**
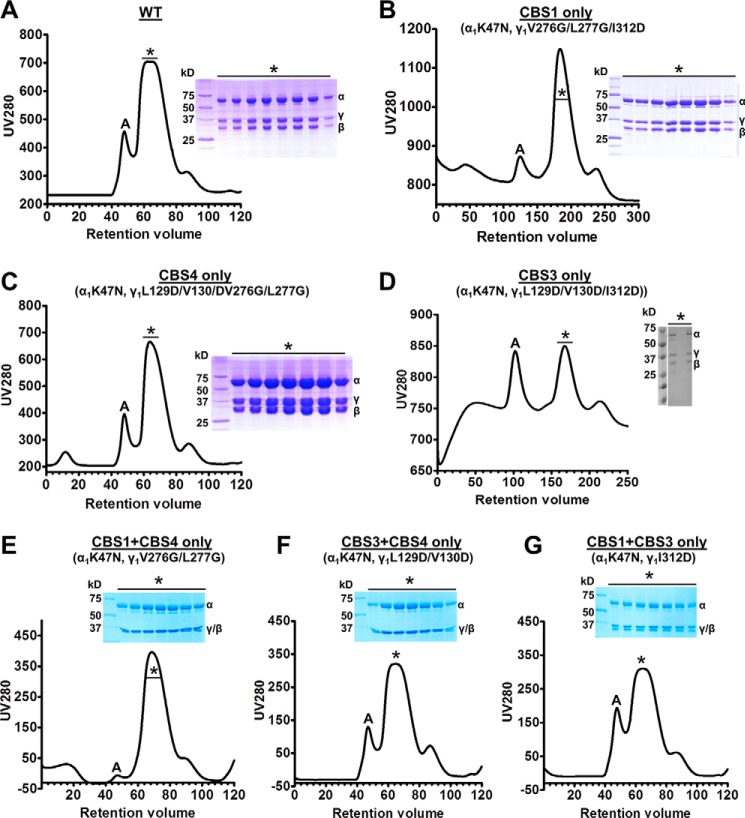
**Size exclusion chromatograms and SDS-PAGE of AMPK mutant proteins.** *A*, *C*, and *E–G*, 120-ml column; *B* and *D*, 300-ml column; *, monomeric α1β2γ1 fractions; *A*, aggregate/oligomers.

**Figure 3.**
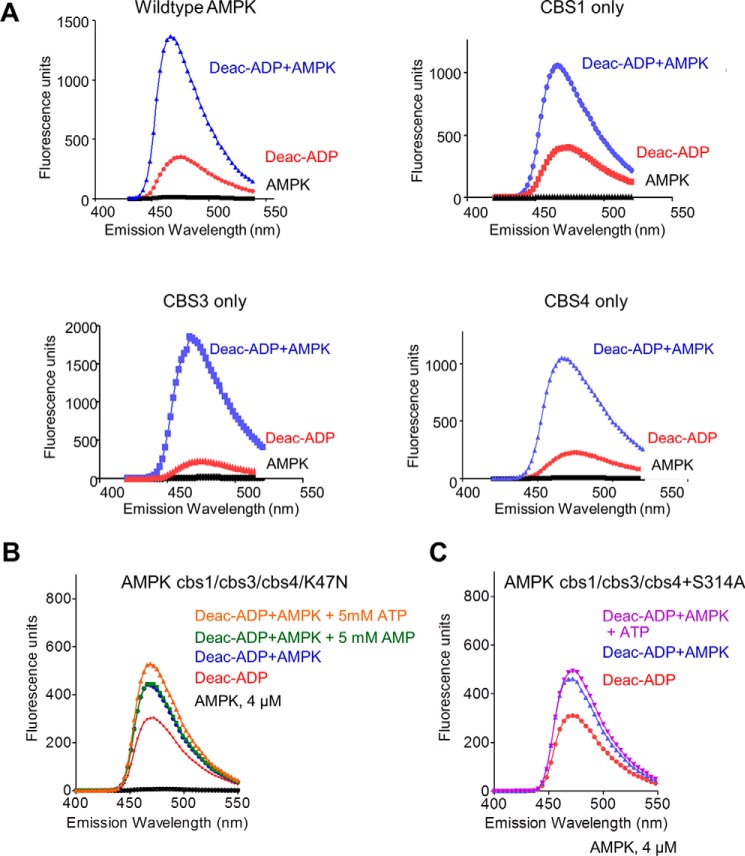
**Fluorescence spectra of wild-type and mutant AMPK/deac-ADP and competition by AMP and ATP.** *A*, emission spectra of 0.5 μm deac-ADP in the absence and presence of wild-type and mutant AMPK proteins in which only one of the CBS sites remained functional (CBS1 only: α1-K47N, γ1-V276G/L277G/I312D; CBS3 only: α1-K47N, γ1-L129D/V130D/I312D; CBS4 only: α1-K47N, γ1-L129D/V130D/V276G/L277G). Excitation: 430 nm. *B* and *C*, emission spectra of 0.5 μm deac-ADP in the absence and presence of 4 μm α1β2γ1 AMPK (α1-K47N, γ1-L129D/V130D/V276G/L277G/I312D) (*B*) or α1β2γ1 AMPK (α1-K47N, γ1-L129D/V130D/V276G/L277G/I312D/S314A) (*C*) and 5 mm AMP or ATP.

The phosphate groups of the three AMP molecules in AMP-bound AMPK face toward each other and are coordinately bound by a set of charged amino acids, whereas the adenine rings face away from each other and are bound by CBS-specific residues (Fig. 1). Specifically, His^151^ binds the phosphate groups of both AMP at CBS1 and CBS4, whereas His^298^ forms hydrogen bonds with the phosphates at both CBS3 and CBS4, suggesting that binding to one CBS motif could affect binding to the other sites. To characterize nucleotide binding at individual CBS motifs in the context of holo-AMPK, but absence of other nucleotide-binding sites, we generated a set of three AMPK variants in which we mutated the ATP-binding lysine in the kinase domain (α_1_-K47N) plus adenine-binding residues previously shown to be required for nucleotide binding (20) at two of three CBS sites, thus only one functional CBS remains (Fig. 1). All mutant proteins were stable and could be purified as stoichiometric complexes from size exclusion chromatography columns (Fig. 2). Similar to a previous approach (19), we determined relative nucleotide affinities by incubating wild-type and mutant proteins with a fluorescently labeled adenine nucleotide analog and competed the interaction with unlabeled AMP or ATP. Incubation of 500 nm 3′-(7-diethylaminocoumarin-3-carbonylamin)-3′-deoxyadenosine-5′-diphosphate (deac-ADP; see “Experimental procedures”) (33) with 4 μm of the recombinant human α_1_β_2_γ_1_-isoform of AMPK resulted in a strong increase in deac-ADP fluorescence and a shift of the emission maximum to a shorter wavelength (Fig. 3*A*), indicating that deac-ADP binds wild-type and the three mutant AMPK proteins.

**Figure 4.**
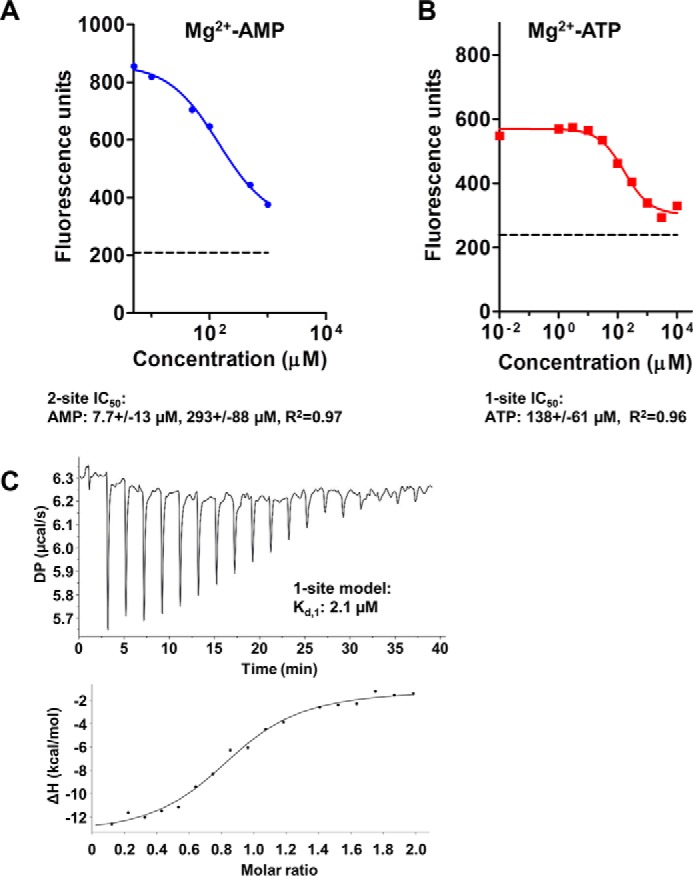
**Nucleotide binding to wild-type AMPK.** *A* and *B*, competition of 0.5 μm deac-ADP bound to α1β2γ1 AMPK by unlabeled Mg^2+^-AMP (*A*) and Mg^2+^-ATP (*B*). The *dashed line* indicates the fluorescence signal of unbound deac-ADP. Because fluorescence competition failed to unambiguously determine the IC_50_ value for the high affinity AMP-binding site, we independently determined its affinity by ITC (*C*).

As controls, we first incubated deac-ADP with AMPK in which the adenine-binding residues at all three CBS sites were mutated (plus K47N in the kinase domain), which resulted in a small increase in fluorescence (Fig. 3*B*). This indicated that deac-ADP, in which the ADP-ribose is modified by a bulky coumarin derivative, has residual AMPK-binding activity. This binding signal could not be competed with a 10,000-fold molar excess of AMP, consistent with the reported inability of AMP to bind to this triple cbs mutant (cbs1, L129D/V130D; cbs2, V276G/L277G; cbs3, I312D) (20). Neither could ATP compete with the increase in fluorescence, but surprisingly caused a minor increase in fluorescence that was diminished by introduction of an additional cbs4 mutation, S314A (Fig. 3*C*). Second, in excellent agreement with previous studies (15, 19, 22), Mg^2+^-AMP bound wild-type AMPK, and competed the binding of deac-ADP to wild-type AMPK, with biphasic kinetics, consistent with two exchangeable binding sites, with *K_d_* values of 2.1 μm (high affinity site) and ∼290 μm (low affinity site) (Fig. 4, *A–C*), whereas Mg^2+^-ATP competed with monophasic kinetics, consistent with similar affinities for both exchangeable sites (Fig. 4*B*).

**Figure 5.**
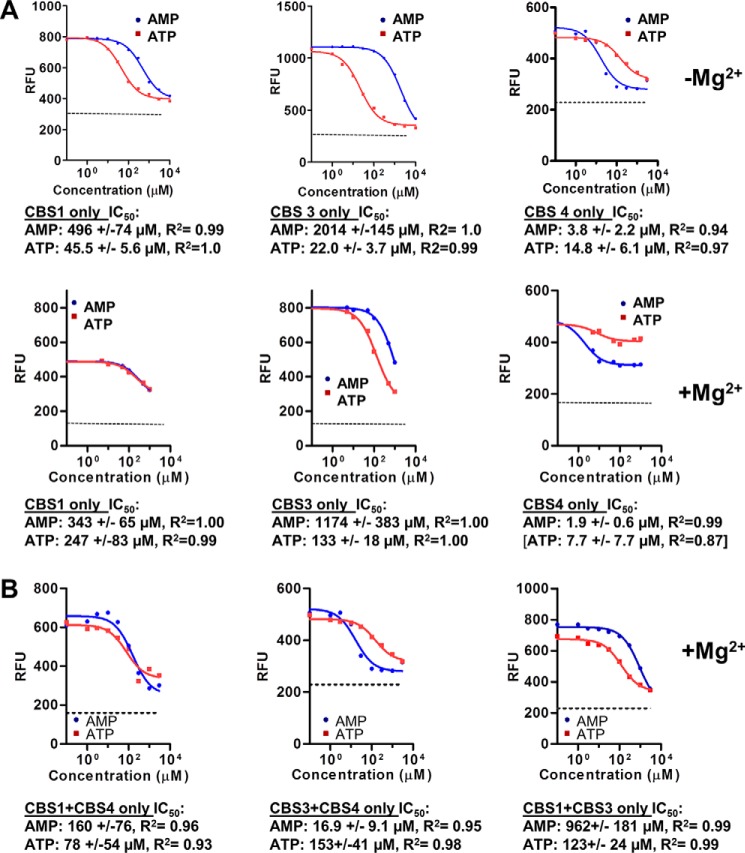
**Nucleotide binding to individual CBSs and pairs of CBSs.** Competition of 0.5 μm deac-ADP bound to 4 μm α1β2γ1 AMPK by unlabeled AMP and ATP. *A*, AMPK mutants with only a single functional adenine nucleotide-binding site (see Fig. 1). Note that in these experiments singly and doubly mutated CBS4 (I312D and I312D/S314A) behaved identically. The ability of Mg^2+^-AMP to compete deac-ADP from 4 μm AMPK with an IC_50_ of 1.9 μm indicates that AMP still binds CBS4 with submicromolar affinity, but no longer non-exchangeably. *B*, AMPK mutants with two functional adenine nucleotide-binding sites. Samples were incubated for 30 min, excited at 430 nm, and emission recorded at 470 nm. The *dashed line* indicates the fluorescence signal of unbound (*i.e.* completely competed) deac-ADP.

We then tested the ability of AMP and ATP to compete deac-ADP from the AMPK mutant proteins. In contrast to wild-type AMPK, the “non-exchangeable” AMP-binding site CBS4 became exchangeable in the cbs1/cbs3 mutant, as it was efficiently bound by deac-ADP, and binding could be competed with IC_50_ values of 3.8 μm (AMP) and 14.8 μm (ATP) in the absence of Mg^2+^, and 1.9 μm for AMP in the presence of Mg^2+^ (Fig. 5*A*; note that Mg^2+^-ATP inefficiently competed deac-ADP from the CBS4 only mutant, resulting in an unreliable IC_50_ value for this measurement). Both AMPK cbs3/cbs4 (CBS1 only) and AMPK cbs1/cbs4 (CBS3 only) bound ATP with low to moderate affinity and AMP with very low affinity. Especially CBS3 in the absence of CBS4 and CBS1 bound AMP with an IC_50_ value that is well below physiological AMP concentrations and with a more than 8-fold preference of ATP over AMP in the presence of Mg^2+^ (and more than 90-fold in its absence). Given that the physiological concentration of ATP is close to 2 orders of magnitude higher than that of AMP, CBS3 would be constitutively bound by ATP in the absence of functional CBS4 and CBS1.

Next we determined binding of AMP and ATP to AMPK in which only the ATP-binding site in the kinase domain and a single CBS site were mutated, leaving intact two binding sites in all three combinations. Importantly, Chen *et al.* (20) had shown that in these mutants CBS4 remains tightly AMP-bound during protein purification. In agreement with this report, when we left CBS3 and CBS4 intact (α1-K47N, γ1-L129D/V130D), AMP and ATP competed a single site (*i.e.* data cannot be modeled to two sites), confirming that the presence of wild-type CBS3 is sufficient to allow CBS4 to bind AMP tightly enough to not noticeably exchange during competition. In the presence of wild-type CBS4, CBS3 bound AMP with relatively high affinity (16.9 μm) and ATP with much lower affinity (153 μm) (Fig. 5*B*), resembling the high affinity exchangeable binding site in the context of wild-type AMPK. In contrast, when we mutated the catalytic site plus CBS3 (CBS1 + CBS4 intact), the competition again fit only a single site model, yet the affinity for AMP remained weak (IC_50_ = 160 μm) and below that of ATP (IC_50_ = 78 μm) (Fig. 5*B*), resembling more closely the low affinity exchangeable AXP-binding site. Finally, when only CBS1 and CBS3 remained functional, we could fit the competition data only as a single site with low affinity for ATP and very low affinity for AMP (Fig. 5*B*), similar to what we have found for the exchangeable low affinity binding site in wild-type AMPK. Collectively, these data indicate that (i) either a functional CBS1 or a functional CBS3 is sufficient to allow CBS4 to bind AMP non-reversibly during competition, that (ii) in the presence of functional CBS4, CBS3 resembles more the high affinity and CBS1 the low affinity exchangeable site, and (iii) that in the presence of only CBS1 and CBS3 neither formed a high affinity binding site, but both most likely formed low affinity binding sites that could not be computationally deconvoluted.

### AXP binding to a single CBS motif modulates the conformation of all three CBS sites as well as the CBM

**Figure 6.**
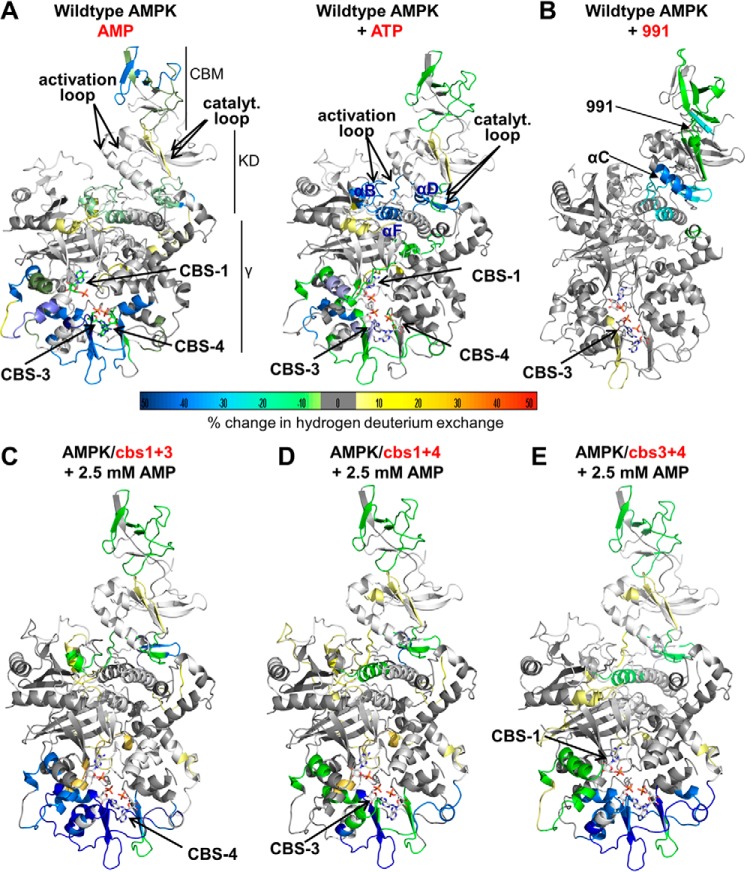
**HDX-MS changes of wild-type and mutant AMPK upon incubation with AMP or ATP.** Changes were overlaid as heat map onto the structure of α1β2γ1 AMPK (4RER). The heat map legend (% change) is shown at the *bottom. A*, AMPK wild-type in the presence *versus* absence of AMP (*left*) or ATP (*right*). *B*, AMPK wild-type in the presence *versus* absence of 991. *C*, *D*, and *E*, AMPK triple mutants in which only CBS4 (*C*), CBS3 (*B*), or CBS1 (*E*) are functional (see Fig. 1).

To directly test conformational stabilization, we analyzed wild-type and mutant AMPK by HDX-MS. HDX-MS measures the backbone amide exchange of hydrogen against deuterium in deuterated buffers. Ligand binding typically protects binding sites against deuterium exchange and can also induce conformational changes that alter protection patterns. Incubation of wild-type AMPK with 2.5 mm AMP dramatically reduced hydrogen/deuterium exchange at CBS3 and CBS4, strongly at CBS1, and moderately at the catalytic center of the kinase domain (catalytic loop, activation loop, and parts of helices αB, αD, and αF; Fig. 6*A* and supplemental Tables S1–S3). Because AMP at CBS4 exchanges very slowly, the strong decrease in HDX at CBS4 is likely predominantly or completely due to its conformational stabilization upon AMP binding to CBS1 and CBS3.

Unexpectedly, the CBM of the β-subunit was also strongly protected (Fig. 6*A*, *left panel*, and supplemental Table S3), indicating that the adenine nucleotide-binding sites in the γ-subunit and the CBM are conformationally connected, even though these domains are located on opposite sides of the AMPK heterotrimer and do not contact each other. Conversely, when we incubated AMPK with the Merck compound 991, which binds a pocket formed by the CBM and kinase domain (allosteric drug and metabolite-binding site) (34), the 991-binding site and the regulatory αC-helix became strongly stabilized, whereas the distal CBS3 in the γ-subunit became mildly destabilized (Fig. 6*B*). AMP-dependent protection against dephosphorylation of *p-*Thr^174^ in the activation loop likely involves occluding *p-*Thr^174^ by the CBM linker (15). The observed CBM stabilization by AMP binding may therefore play a direct role in this critical AMP response. When we incubated AMPK with ATP, the overall protection was similar to that with AMP. However, protection of CBS3, CBS4, and the CBM was weaker, whereas protection of CBS1 and the ATP-binding catalytic site in the kinase domain increased (Fig. 6*A*, *right panel*, and supplemental Tables S1–S3).

When we incubated the AMPK mutant protein in which CBS4 is the only functional AXP-binding site (K47N/cbs1/cbs3) with AMP, CBS4 became strongly protected, confirming that CBS4 is indeed exchangeable in the absence of CBS1 and CBS3. In addition, CBS3 became protected, indicating that AMP-binding at CBS4 strongly conformationally stabilizes CBS3 (Fig. 6*C*), in excellent agreement with the fluorescence competition data. In contrast to CBS3, AMP-binding at CBS4 modulated accessibility at CBS1 more mildly (Fig. 6*C*; note that deuterium exchange both increases and weakly decreases in different CBS1 peptides), consistent with the relatively small effect of CBS4 on CBS1 AMP-binding. A similar, but milder protection of CBS3 and CBS4 occurred for the CBS3-only (K47N/cbs1/cbs4) AMPK mutant in the presence of AMP (Fig. 6*D*), whereas AMP-binding to the CBS1-only (K47N/cbs3/cbs4) AMPK mutant stabilized all three binding sites (Fig. 6*E*).

### CBS occupancy in mixtures of adenine nucleotides

**Figure 7.**
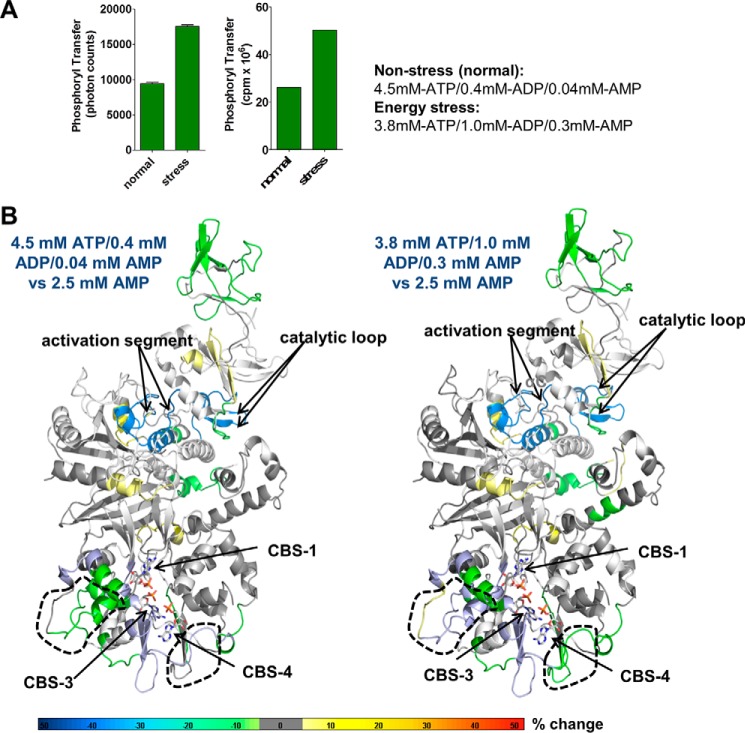
**Changes in HDX-MS protection in the presence of AXP mixtures mimicking non-stress and energy stress conditions.** *A*, AMPK kinase assay in the presence of AXP mixtures mimicking nonstress (4.5 mm ATP, 0.4 mm ADP, 0.04 mm AMP) and energy stress (3.8 mm ATP, 1.0 mm ADP, 0.3 mm AMP) conditions. *Left panel*, AlphaScreen-based kinase assay; *right panel*, radioactive kinase assay. *B*, HDX-MS perturbation map of AMPK in the presence of the two different AXP mixtures. HDX-MS changes were overlaid as heat map onto the structure of α1β2γ1 AMPK (PDB code 4RER). Main areas of differential protection are highlighted by *dashed outlines*. Note that there is no change at the catalytic site, indicating that the catalytic site is constitutively bound by ATP as expected. The color code (% change) is shown below the structures. The relatively mild changes are consistent with noticeable ATP exchange only at CBS3, whereas the non-physiological shift from high concentration of pure AMP to high concentration of pure ATP resulted in strong HDX changes (Fig. 6*A*), consistent with near full nucleotide exchange at both CBS3, CBS1, and ATP binding/release at the catalytic site.

**Figure 8.**
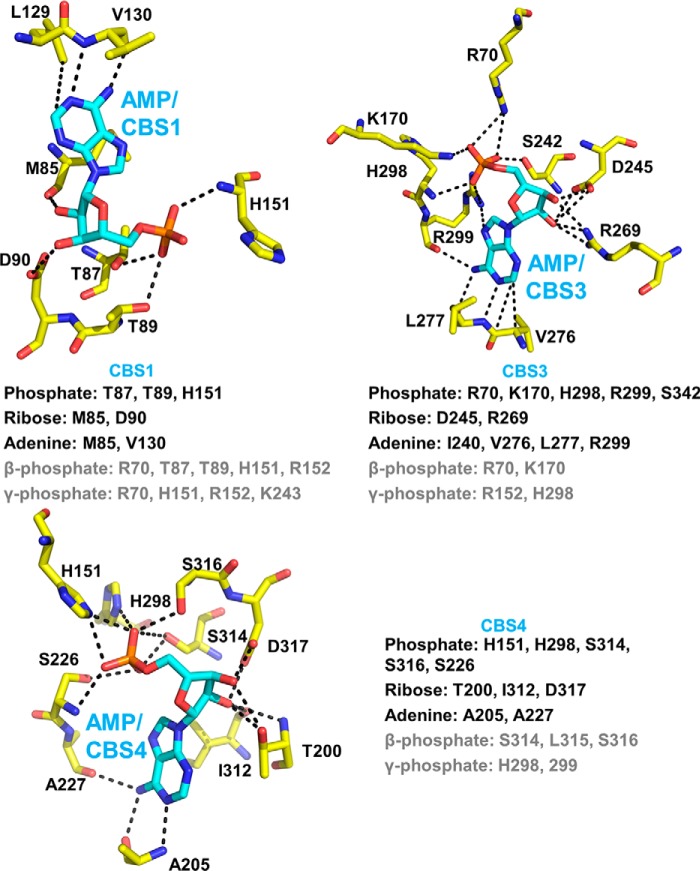
**AXP/CBS interaction network in human AMPK-γ1.** Shown are the three AMP-bound CBS sites in holo-AMPK co-crystallized with AMP. β- and γ-phosphate interacting residues (*gray*) are collectively based on the structures of core AMPK co-crystallized with ATP (PDB code 4EAG) (20) and core- AMPK soaked with ATP (PDB codes 2V92 and 4EAJ) (19, 20).

All current binding and structural studies have been performed either in the presence of high concentrations of AMP or high concentrations of ATP, whereas in cells AMPK is exposed to mixtures of AMP, ADP, and ATP. An important, but unresolved, question is therefore which CBS motifs are occupied by AMP, ADP, or ATP under physiological conditions, with AMP always present, but at levels that are 10–100-fold lower than ATP levels. To probe for occupancy under physiological conditions, we incubated AMPK with two different AXP mixtures: 4.5 mm ATP, 0.4 mm ADP, 0.04 mm AMP and 3.8 mm ATP, 1.0 mm ADP, 0.3 mm AMP, which have been reported to mimic normal cellular (non-stressed) and energy stress conditions in cells, respectively (14). Consistently, the AXP ratio mimicking energy stress allosterically activated recombinantely purified AMPK 2-fold relative to the mixture mimicking energy excess (Fig. 7*A*), the same fold direct activation as for the standard switch from AMPK, 100 μm ATP to AMPK, 100 μm ATP + 200 μm AMP. In striking contrast to the different HDX profiles of AMPK, 2.5 mm AMP *versus* AMPK, 2.5 mm ATP (Fig. 6, *A* and *B*, and supplemental Tables S1–S3), the profiles in the presence of the stress and non-stress AXP mixtures were quite similar to each other and showed significant differences only in two small areas (Fig. 7*B*), clearly indicating that these conditions induce much more limited nucleotide exchange. Whereas HDX-MS has the potential to distinguish AMP *versus* ATP occupancy due to the unique β- and γ-phosphate groups of ATP, none of the resolved β- and γ-phosphate contacting peptides makes exclusive interactions with these groups (see Fig. 8 and supplemental Table S1) and decreases in HDX can be due to both direct nucleotide binding and to conformational stabilizations. Because our fluorescence competition data are most consistent with CBS1 (binds the more abundant ATP with equal or higher affinity than AMP) being bound by ATP under both normal and energy stress conditions, and CBS4 being essentially non-exchangeably bound to AMP, only CBS3 may undergo noticeable ATP/AMP exchange at physiological levels.

### NADPH binds AMPK at the high affinity exchangeable AXP-binding site

**Figure 9.**
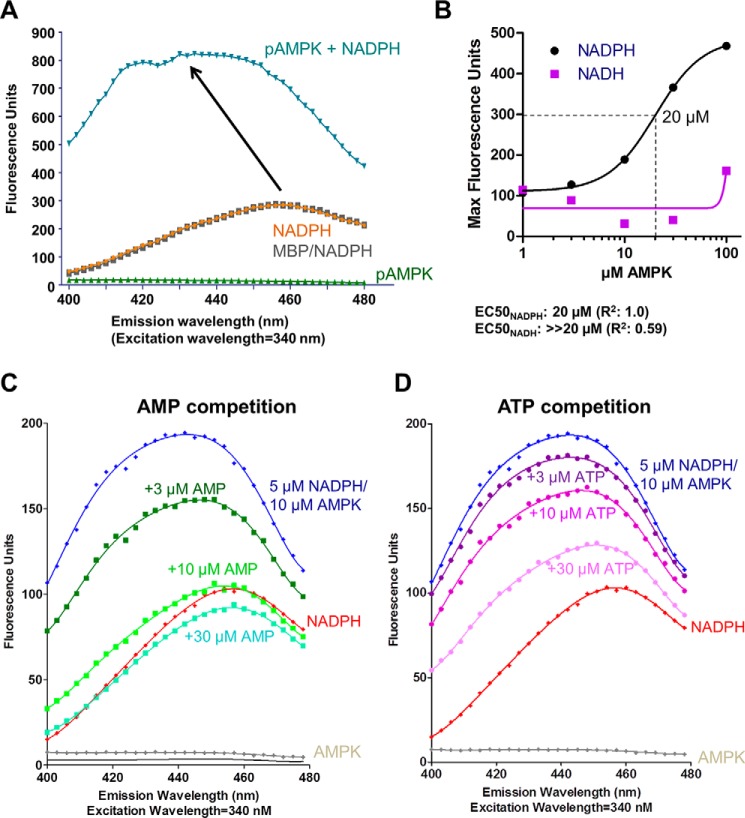
**NADPH binds adenine nucleotide-binding site(s) in the γ-subunit.** *A*, NADPH emission spectra in the absence and presence of MBP and MBP-tagged phosphorylated α1β2γ1 AMPK. The spectrum of AMPK in the absence is shown as negative control. *B*, estimation of NADPH and NADH binding constants. 5 μm NADPH or NADH were incubated with increasing concentrations of MBP-AMPK and MBP-AMPK concentrations were plotted against fluorescence intensity at the emission maxima. *C* and *D*, AMP and ATP compete the NADPH binding signal. Emission spectra of 5 μm NADPH, 10 μm AMPK were recorded in the absence and presence of increasing concentrations of AMP (*C*) and ATP (*D*).

We found that in addition to the known AMPK ligand NADH (15), NADPH can also bind AMPK. When we incubated 5 μm NADPH with 10 μm maltose-binding protein (MBP)-tagged AMPK, NADPH fluorescence increased and shifted its emission maximum, indicative of direct binding (Fig. 9*A*). In contrast, incubation of NADPH with 10 μm of the MBP control did not alter the NADPH emission spectrum (Fig. 9*A*). To estimate the affinity for NADPH and NADH, we incubated 5 μm NADPH or 5 μm NADH with increasing concentrations of MBP-AMPK. Although the half-maximal increase of the NADPH fluorescence signal indicated a *K_d_* of ≤20 μm, an increase of NADH fluorescence did not approach saturation at the highest AMPK concentration (100 μm), indicating that NADH binds AMPK only with low affinity (≫20 μm; Fig. 9*B*). Because NADPH is an adenine dinucleotide, we speculated that it would bind to one or more of the AXP-binding sites in the γ-subunit. Indeed 10 μm AMP almost completely competed 5 μm NADPH bound to 10 μm AMPK, indicating that NADPH binds to the high affinity exchangeable AMP-binding site (Fig. 9*C*). Conversely, ATP was also able to compete NADPH, but only at much higher concentrations (Fig. 9*D*). We therefore conclude that NADPH binds to the high affinity exchangeable AMP-binding site and that this site has a preference for AMP over ATP, suggesting that NADPH binds CBS3.

**Figure 10.**
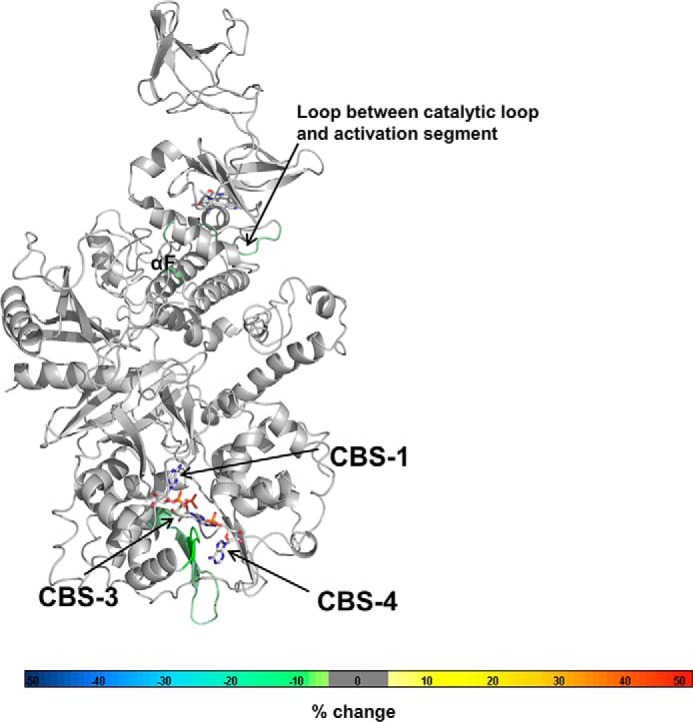
**Binding of NADPH protects CBS3 and CBS4.** HDX-MS changes wild-type AMPK upon incubation with 1 mm NADPH. Changes were overlaid as heat map onto the structure of α1β2γ1 AMPK (PDB code 4RER). The heat map legend (% change) is shown at the *bottom*.

To physically localize the NADPH-binding site, we collected HDX-MS perturbation data of AMPK in the absence *versus* presence of 1 mm NADPH. As shown in Fig. 10, NADPH binding selectively protected CBS3 (plus CBS4), but not CBS1, against deuterium exchange. Because CBS4 is non-exchangeable, the HDX-MS data confirm that NADPH specifically binds CBS3. Given the high affinity (low μm IC_50_) with which AMP competes NADPH from CBS3 and the AMP-over-ATP binding preference of CBS3, we conclude that CBS3, and not as previously proposed CBS1 (19), is the high affinity exchangeable AMP-binding site.

## Discussion

AMPK plays critical roles in numerous aspects of physiology (4, 5, 35) and is an important target for the treatment of metabolic diseases, including diabetes and obesity (1, 7), as well as cancer (2, 6, 36) and heart disease (37). In addition, AMPK activation mimics many of the beneficial effects of exercise and caloric restriction (38–41). Crystal structures and biochemical binding assays have demonstrated that adenine nucleotides can competitively bind to three functional CBS motifs in the γ-subunit, of which CBS3 functions as an adenine sensor site. However, due to the technical difficulties of deconvoluting complex binding events, many aspects of AXP occupancy at individual CBS sites, and how binding to one site is communicated to the other sites, have remained unclear.

To deconvolute the binding events, we generated a set of mutant AMPK complexes in which only individual sites or pairs of sites remain functional, and quantitatively determined their nucleotide-binding and deuterium-exchange patterns. These experiments provided insight into functional and conformational coupling of CBS sites, occupancy in the presence of AXP mixtures, assignment of the high affinity exchangeable AXP site, and a mechanistic model for the requirement of CBS4 for AMP sensing at CBS3.

### Functional and conformational coupling of CBS sites

Early binding studies using GST-CBS fusion proteins provided the first support for cooperative AXP binding (18). Here we specifically demonstrate that CBS4 becomes readily exchangeable in the absence of functional CBS1 and CBS3, and AXP-binding to either CBS1 or CBS3 strongly decreased deuterium exchange at CBS4. Therefore, AXP-bound CBS1 and CBS3 can each separately increase AMP affinity at CBS4 and conformationally stabilize CBS4. Consistently, AMPK with mutation of only CBS1 or only CBS3 stably co-purifies with AMP (3) and AXP-binding follows a single site binding model with kinetics different from that of CBS4-only. Stabilization is likely due to His^151^, which forms hydrogen bonds with the AMP phosphate groups at both CBS4 and CBS1, and His^298^, which forms hydrogen bonds with the AMP phosphate groups at both CBS4 and CBS3 (Fig. 1). Conversely, CBS3 becomes conformationally stabilized by CBS4 and CBS1, and AMP-binding to CBS4 (functional CBS3 + CBS4 *versus* functional CBS3) increased the CBS3 affinity for AMP by 2 orders of magnitude and increased the AMP/ATP binding preference by 2–3 orders of magnitude.

### AMP-sensing requires CBS4 to conformationally stabilize AMP-bound CBS3

Mutating a key adenine-binding residue of CBS4 (I312D) abolishes direct allosteric AMPK activation by AMP (20). This indicates that CBS4 critically contributes to AMP-sensing at CBS3/αRIM, even though CBS4 is constitutively bound by AMP and does not directly interact with either the αRIM loop or AMP at CBS3. Our fluorescence competition and HDX-MS data suggest that CBS4 is required for AMP sensing by stabilizing AMP binding at CBS3, which increases both the affinity for AMP and the preferential binding of AMP over ATP (Fig. 5). AMP-bound CBS4 therefore allows AMP exchange at CBS3 at nucleotide concentrations that reflect cellular conditions of energy excess ([ATP]/[AMP] >100) and energy stress ([ATP]/[AMP] ∼10–20). Our finding that AMP-bound CBS4 selectively stabilizes AMP binding at CBS3 provides a rationale for the CBS4 requirement for AMP sensing.

**Figure 11.**
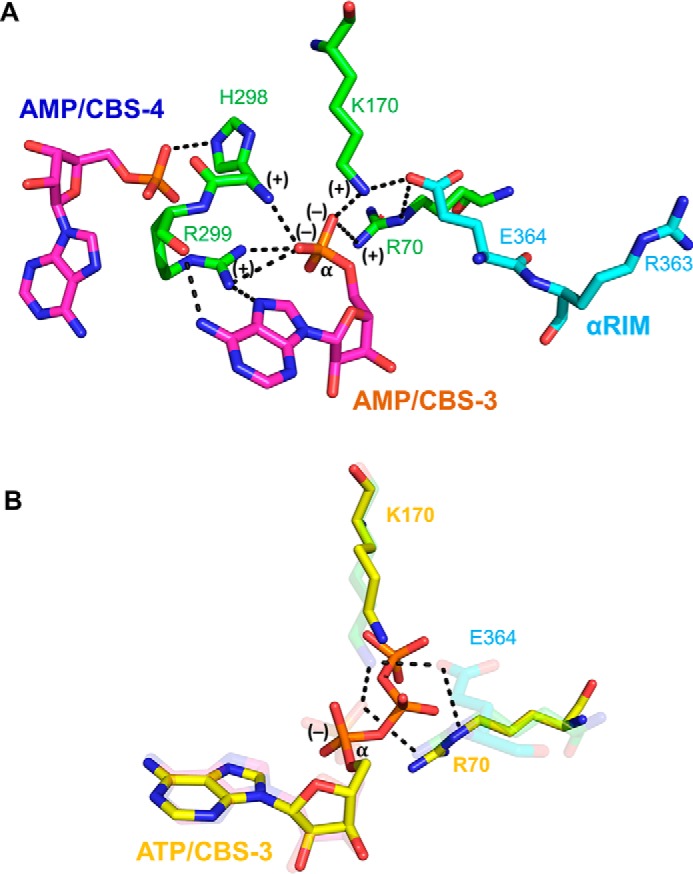
**CBS4 and αRIM stabilize AMP-binding at CBS3 and increase the AMP/ATP-binding preference.** *A*, CBS4-CBS3-αRIM network in AMP-bound AMPK (PDB code 4CFE). *B*, transparent structure from *panel A* overlaid with ATP, Arg^70^, and Lys^170^ from the structure of ATP-soaked core-AMPK (PDB code 2V92). Note that the α-phosphate of ATP has only a single negative charge and that the β- and γ-phosphate groups of ATP push Lys^170^ and Arg^70^ away from αRIM Glu^364^.

How does AMP binding to CBS4 mechanistically increase AMP affinity and AMP/ATP-binding preference at CBS3? Inspection of AMPK crystal structures indicates that stabilization is predominantly due to CBS4 positioning the side chain of His^298^. The His^298^ backbone amide directly forms a hydrogen bond with the phosphate group of AMP at CBS3 and positions the adjacent key residue Arg^299^, which forms two hydrogen bonds with the AMP phosphate group and two with the AMP adenine ring at CBS3 (Fig. 11). Similarly, αRIM Glu^364^ positions CBS3 Arg^70^ and Lys^170^, which form salt bridges with the phosphate group of AMP at CBS3. The phosphate group of AMP carries two negative charges to interact with the three positively charged residues stabilized by αRIM (Arg^70^ and Lys^170^) and CBS4 (Arg^299^) plus the partially positive backbone amide of His^298^. In contrast, the corresponding α-phosphate of ATP carries only a single charge and therefore interacts less tightly with the positive charge cluster (Fig. 11*A*). In addition, as seen when structurally aligned with ATP-soaked core-AMPK (PDB code 2V92), the side chains of Arg^70^ and Lys^170^ need to move away in opposite directions from Glu^364^ to avoid clashes with the β- and γ-phosphate groups of ATP (Fig. 11*B*), thereby weakening the Glu^364^ interaction. Together, these constraints provide a structural explanation for both CBS4 and αRIM Glu^364^ collectively stabilizing AMP preferentially over ATP at CBS3. Consistent with a requirement of both CBS4 and the αRIM for high affinity binding of AMP at CBS3, AMP was preferentially bound to CBS1 and CBS4 in the crystal structure of the αRIM-lacking core-AMPK (19), but preferentially bound to CBS3 and CBS4 in the context of the αRIM-containing holo-AMPK (15, 42).

### CBS3 is the high affinity exchangeable AMP-binding site

Our work presents several lines of evidence in support of CBS3 being the high affinity exchangeable AMP-binding site. First, AMP competes NADPH binding with high affinity, whereas ATP competes with moderate affinity, directly indicating that the NADPH-binding site is the high affinity site. This site is CBS3, as NADPH selectively protects CBS3, but not CBS1, against hydrogen deuterium exchange. Second, CBS3 in the presence of functional CBS4 and αRIM binds AMP with high, and ATP with moderate affinity, whereas CBS1 in the presence of functional CBS4 binds AMP weakly and with preference of ATP over AMP binding. Third, HDX-MS in the presence of wild-type holo-AMPK demonstrates that AMP stabilizes CBS3 more strongly than ATP, whereas it stabilizes CBS1 less strongly than ATP, consistent with higher affinity AMP binding at CBS3 and relatively stronger ATP binding at CBS1.

### Under physiological conditions nucleotide exchange is likely limited to CBS3

Several lines of evidence suggest that during the shift from non-stress (4.5 mm ATP, 0.4 mm ADP, 0.04 mm AMP) to energy stress (3.8 mm ATP, 1.0 mm ADP, 0.3 mm AMP) only CBS3 noticeably exchanges ATP against AMP. By mutating CBS1, we could show that in the presence of the tightly AMP-bound CBS4, CBS3 binds AMP with 10- to 100-fold higher affinity than ATP, closely matching the 10- to 100-fold higher ATP to AMP levels under different stress conditions. In contrast, under the converse conditions, CBS1 binds ATP with similar or higher affinity than AMP, suggesting that at millimolar ATP and micromolar AMP physiological levels CBS1 remains largely ATP-bound and CBS4 AMP-bound. These conclusions are supported by HDX-MS. First, the AMP/ATP preferences of CBS3 and CBS1 are reflected by CBS3 being more strongly protected by AMP than ATP and, conversely, CBS1 is more strongly protected by ATP than AMP. Second, the AMPK HDX profiles in the sole presence of AMP *versus* ATP differ strongly, consistent with AMP/ATP exchange at both CBS1 and CBS3 as seen in crystal structures and competition assays, whereas the profiles in the presence of stress *versus* non-stress AXP levels are much smaller, indicating reduced AMP/ATP exchange.

**Figure 12.**
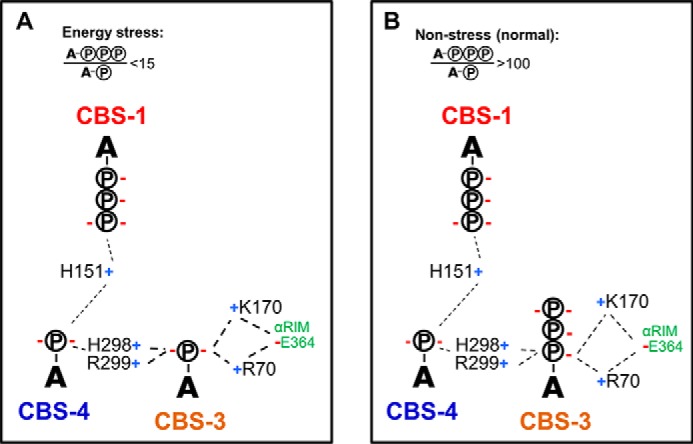
**Schematic model of AXP occupancy under energy-stress (*A*) and non-stress (*B*) conditions.** Positive and negative charges are indicated by *blue* “+” and *red* “−” signs. *A-circled P*, AMP; *A-3 circled P*, ATP.

ATP is the essential cofactor of numerous cellular reactions, which require ATP concentrations within a very tight range. Yet ATP is constantly and rapidly metabolized (humans hydrolyze roughly their own body weight of ATP per day (43)), and therefore requires highly efficient cellular and organismal regulation of ATP homeostasis. This task is centrally performed by AMPK and depends on the ability of AMPK to precisely and sensitively detect AXP ratios under different physiological conditions to modulate its kinase activity. Our study, together with previous binding assays and crystal structures of holo-AMPK bound to AMP and core-AMPK bound to ATP, provides a first view of the intricate arrangement and interactions between nucleotide-binding sites to allow precise and highly sensitive sensing of AXP ratios under physiological stress and non-stress conditions (Fig. 12). Understanding how the sensed nucleotides modulate kinase activity and activation loop accessibility will require the missing key structure of holo-AMPK in its inactive ATP-bound state.

## Experimental procedures

### DNA constructs and reagents

The human wild-type His_6_-α_1_(11–550)β_2_γ_1_ AMPK expression construct was described previously (32). Mutations were introduced by site-directed mutagenesis using the QuikChange method (Stratagene) or standard PCR-based methods. All expression constructs and mutations were confirmed by DNA sequencing.

### Protein expression and purification

Expression plasmids were transformed into *Escherichia coli* BL21(DE3). Cells were grown to an *A*_600_ of ∼1 at 28 °C and induced with 100 μm isopropyl β-d-thio-galactopyranoside at 16 °C overnight. Cell pellets were resuspended in 25 mm Tris, pH 8.0, 300 mm NaCl, 25 mm imidazole, 10% glycerol, 5 mm β-mercaptoethanol, and lysed by French Press with pressure set to 900 pascal. Lysates were cleared by centrifugation for 30 min at 20,000 × *g*, passed over a 50-ml nickel-chelating HP-Sepharose column (GE Healthcare), and eluted with 25 mm Tris, pH 8.0, 300 mm NaCl, 500 mm imidazole, 10% glycerol, 5 mm β-mercaptoethanol. The eluted His-tagged AMPK was further purified by size-exclusion chromatography through a 300-ml HiLoad 26/60 Superdex 200 column (GE Healthcare) in 10 mm Tris, pH 8.0, 150 mm NaCl, 5 mm MgCl_2_, 1 mm EDTA, 10% glycerol, 2 mm DTT. The proteins eluted from the gel filtration column at a volume corresponding to the size of a monomeric complex at a purity ≥95% as judged by SDS-PAGE (Fig. 2).

### Fluorescence binding and competition assays

Deac-ADP was synthesized by the method of Webb *et al.* (44). 4 μm AMPK wild-type and mutant proteins were incubated with 0.5 μm deac-ADP in 10 mm Tris, pH 8.0, 150 mm NaCl, 1 mm EDTA, 10% glycerol, 2 mm DTT in the presence and absence of 5 mm MgCl_2_ for 30 min at room temperature. The emission spectrum of deac-ADP was recorded using a SpectraMax M2^e^ reader (Molecular Devices Corporation). For deac-ADP competition assays, AMP or ATP were added at increasing concentrations, whereas deac-ADP was kept at a constant concentration (0.5 μm). The IC_50_ values were obtained from curve fitting to the competitive inhibitor model using GraphPad Prism.

For NADPH emission spectra, 5 μm NADPH were incubated with either 10 μm MBP or 10 μm MBP-AMPK and excited at 340 nm using the SpectraMax M2^e^ cuvette mode. For binding isotherms, NADP and NADPH (5 μm each) were incubated with increasing concentrations of AMPK, excited at 340 nm, and full emission spectra were recorded. Fluorescence emission maxima were plotted against AMPK concentration and EC_50_ values were obtained from curve fitting to the one-site binding model using GraphPad Prism.

### HDX coupled with mass spectrometry

Solution-phase amide HDX experiments were carried out using a fully automated system as described previously (45, 46). Five microliters of a 10 μm protein solution were mixed with 20 μl of D_2_O containing HDX buffer (20 mm KPO_4_, pH 7.4, 50 mm KCl) and incubated at 4 °C for 10, 30, 60, 300, 900, and 3,600 s. Following on exchange, unwanted forward or back exchange was minimized and the protein was denatured by dilution with 25 μl of quench solution (0.1%, v/v, trifluoroacetic acid (TFA) in 3 m urea). Samples were then passed through an immobilized pepsin column (prepared in house) (47) at 200 μl/min (0.1%, v/v, TFA, 15 °C) and the resulting peptides were trapped on a C8 trap column (Hypersil Gold, Thermo Fisher). The bound peptides were then gradient eluted (4–40%, w/v, CH_3_CN and 0.3%, w/v, formic acid) across a 2 × 50-mm C18 high performance liquid chromatography column (Hypersil Gold, Thermo Fisher) for 5 min at 4 °C. The eluted peptides were then subjected to electrospray ionization directly coupled with a high resolution Orbitrap mass spectrometer (Exactive, Thermo Fisher). Each HDX experiment was carried out in triplicate. Peptide ion signals were confirmed if they had a MASCOT score of 20 or greater and had no ambiguous hits using a decoy (reverse) sequence in a separate experiment using a 60-min gradient. The intensity-weighted average *m*/*z* value (centroid) of each isotopic envelope of the peptide was calculated with in house HDX Workbench software (48) and corrected for back-exchange on an estimated 70% recovery and accounting for the known deuterium content of the on-exchange buffer.

### Isothermal titration calorimetry (ITC)

ITC was performed in a MicroCal PEAQ iTC200 (Malvern) with 18 injections at room temperature. 30 μm AMPK was loaded into the cell and 300 μm AMP in the syringe. Data analysis was performed with the MicroCal PEAQ-ITC instrument control software and yielded: *n* = 0.856 ± 0.02 sites, *K_d_* = 2.06 ± 0.538 μm, Δ*H* = 12.6 ± 0.757 kcal/mol, Δ*G* = −7.76, −*T*Δ*S* = 4.88 kcal/mol.

### Kinase assays

For the radioactive kinase assay, 10 nm phosphorylated AMPK were incubated with 15 μm biotin-SAMS peptide, 2 mm DTT, and 0.25 μl [γ-^32^P]ATP per 15-μl reaction in kinase buffer (25 mm Tris, pH 7.4, 12 mm MgCl_2_, 1 mm Na_3_VO_4_, 5 mm NaF) in the presence of AXP mixtures mimicking non-stress (4.5 mm ATP, 0.4 mm ADP, 0.04 mm AMP) and energy-stress (3.8 mm ATP, 1.0 mm ADP, 0.3 mm AMP) conditions for 30 min at room temperature. Reactions were terminated by addition of 0.5 volumes of 7.5 m guanidine hydrochloride solution in water and reactions were spotted on a SAM 2® Biotin Capture Membrane (Promega). The membrane was washed once for 30 s with 2 m NaCl, 3 times for 2 min with 2 m NaCl, 4 times for 2 min with 2 m NaCl in 1% H_3_PO_4_, and 2 times for 30 s with deionized water to remove unbound reaction components. After drying the membrane at room temperature for 30–60 min, signals were quantitated by phosphorimager analysis.

The luminescence-proximity AlphaScreen-based kinase assay will be described in detail in a future publication and is based on a previously described AMPK activity reporter assay (49). Briefly, 10 nm phosphorylated AMPK were incubated with 50 μm of a biotinylated peptide substrate (biotin-GSTKMRRVATLVDLGYKK) on ice for 10 min. The reaction was terminated by 1000-fold dilution in AlphaScreen buffer (50 mm MOPS, pH 7.4, 50 mm NaF, 50 mm CHAPS, and 0.1 mg/ml of bovine serum albumin) and further incubated in the presence of 50 nm His_6_-Rad53 FHA domain protein, 5 μg/ml each of AlphaScreen streptavidin donor beads and nickel-acceptor beads at room temperature for 90 min in the presence of AXP mixtures mimicking non-stress (4.5 mm ATP, 0.4 mm ADP, 0.04 mm AMP) or energy-stress (3.8 mm ATP, 1.0 mm ADP, 0.3 mm AMP) conditions. Donor beads contain a photosensitizer, which can convert ambient oxygen into short-lived singlet oxygen upon light activation at 680 nm. When the acceptor beads are brought close enough to the donor beads by interaction between His_6_-tagged FHA domain protein and AMPK-phosphorylated biotinylated peptide substrate, singlet oxygen can diffuse from the donor to the acceptor beads and transfer energy to the thioxene derivatives of the acceptor beads, resulting in light emission at 520 to 620 nm, which was measured in an Envision (PerkinElmer Life Sciences) plate reader.

## Author contributions

X. G., Y. Y., S. J. N., A. K., D. G., J. K., M. H. E. T., P. W. deW., L. W., and X. L. conducted the experiments, M. R. W., P. R. G., H. E. X., and K. M. analyzed the results, and K. M. wrote the paper with comments from all authors.

## Supplementary Material

Supplemental Data

## Footnotes

2 The abbreviations used are:

AMPK: AMP-activated protein kinase
αRIM: α-regulatory subunit interaction motif
AXP: AMP/ADP/ATP adenine nucleotides
CBM: carbohydrate-binding module
CBS: cystathionine β-synthetase
deac-ADP: 3′-(7-diethylaminocoumarin-3-carbonylamin)-3′-deoxyadenosine-5′-diphosphate
HDX-MS: hydrogen deuterium exchange mass spectrometry
ITC: isothermal titration calorimetry
MBP: maltose-binding protein
PDB: Protein Data Bank.
