# Supplementary material for: Deconvoluting AMP-activated protein kinase (AMPK) adenine nucleotide binding and sensing
Source: J Biol Chem. 2017 Jun 14;292(30):12653–66. doi: 10.1074/jbc.M117.793018 (PMC5535039; doi:10.1074/jbc.M117.793018)
Supplement: Supplemental Data [file supp_292_30_12653__index.html]

Deconvoluting AMP-dependent kinase (AMPK) adenine nucleotide binding and sensing — Deconvoluting AMP-activated protein kinase (AMPK) adenine nucleotide binding and sensing — Deconvoluting AMPK adenine nucleotide binding and sensing — Supplemental Data 

# Deconvoluting AMP-activated protein kinase (AMPK) adenine nucleotide binding and sensing

## Supplemental Data

- Supplemental Table 2 (.pdf, 108 KB) - HDX-MS protection summary of key AXP binding residue-containing peptides.
- Supplemental Table 1 (.pdf, 289 KB) - Complete HDX-MS peptide list for the gamma 1-subunit.
- Supplemenal Table 3 (.pdf, 314 KB) - HDX-MS peptide list for the ????1- and ????2-subunits
