# Supplementary material for: Deconvoluting AMP-activated protein kinase (AMPK) adenine nucleotide binding and sensing
Source: J Biol Chem. 2017 Jun 14;292(30):12653–66. doi: 10.1074/jbc.M117.793018 (PMC5535039; doi:10.1074/jbc.M117.793018)
Supplement: Supplemental Data [file 10.1074_M117.793018_jbc.M117.793018-1.pdf]

Supplemental Table 2: HDX-MS protection summary of key AXP binding residue-containing peptides. Numbers indicate %ΔHDX upon incubation of purified AMPK with indicated adenine nucleotide(s). Listed are MS-resolved peptides that contain the indicated AXP-binding residues.

		**Wildtype holo-AMPK ($\alpha_1\beta_2\gamma_1$)**				α1-K47G, γ1-L129D/V130D/V276G/L277G **(CBS4 only)**	α1-K47G, γ1-L129D/V130D/I312D **(CBS3 only)**	α1-K47G, γ1-V276G/L277G/I312D **(CBS1 only)**
**CBS**	**Sub-site**	**2.5 mM ATP**	**2.5 mM AMP**	**40 μM AMP**<br>400 μM ADP<br>4.5 mM ATP	**300 μM AMP**<br>1.0 mM ADP<br>3.8 mM ATP	**2.5 mM AMP**		
**CBS-1**	**β-P: T87, T89**<br>**Ribose: D90**	**-9**	+4	-13	-15	**+16**	**+14**	**-3**
	**Adenine:** K127, **L129, V130**	**-6**	0	-3	-4	**N/A**	**N/A**	-2
	α-P: H151<br>γ-P: R152	N/A	N/A	N/A	N/A	N/A	N/A	N/A
**CBS-3**	**Ribose: D245**	-2	**-16**	-6	-7	**-18**	-7	**-13**
	**Adenine:** I240	-7	**-17**	-11	-11	**-21**	-11	**-19**
	**Adenine:** V297	-6	**-9**	-7	-9	**2**	-1	**1**
	**Adenine: V276+L277**	N/A	N/A	N/A	N/A	**-20**	N/A	**-17**
	**α-P: R299**	-6	-9	-7	-9	2	-1	1
	**α-P: K170**	-1	0	-1	-1	N/A	1	0
**CBS-4**	**β-P: L315+S316**<br>**Ribose: D317**	1	-1	0	0	-1	-1	-2
	**Ribose: T200**,	-8	N/A	-10	-8	**-31**	**-15**	**-24**
	**Adenine: A205**	-7	**-16.5**	-10.5	-10.5	**-20.5**	-4	-12
	**Adenine: I312**;	-3	**-8**	-4	-3	**-9**	N/A	N/A
	**Adenine: V225**<br>**α-P: S226**	-13	**-33.5**	-14	-16	**-29**	-6	-15
	α-P: H151	N/A	N/A	N/A	N/A	N/A	N/A	N/A
	**γ-P: R299**	-6	-9	-7	-9	2	-1	1
