# Supplementary material for: Deconvoluting AMP-activated protein kinase (AMPK) adenine nucleotide binding and sensing
Source: J Biol Chem. 2017 Jun 14;292(30):12653–66. doi: 10.1074/jbc.M117.793018 (PMC5535039; doi:10.1074/jbc.M117.793018)
Supplement: Supplemental Data [file 10.1074_M117.793018_jbc.M117.793018-2.pdf]

Supplemental Table 1: Complete ΔHDX-MS peptide list for the γ1-subunit. Resolved peptides are listed in the first column and % changes in HDX are indicated by numbers and heat map color.

Sequence	Charge	Start	END	WT α1β2γ1 AMPK without added nucleotides versus 2.5 mM AMP	WT α1β2γ1 AMPK without added nucleotides versus 2.5 mM ATP	WT α1β2γ1 AMPK without added nucleotides versus Non-stress AXP	WT α1β2γ1 AMPK without added nucleotides versus Stress AXP	CBS4 only + 2.5 mM AMP	CBS3 only + 2.5 mM AMP	CBS1 only + 2.5 mM AMP
NEHPQETPESNNSVY	2	15	29	N/A	N/A	N/A	N/A	6 (3)	5 (4)*	N/A
MKSHRCYDLIPTSSKL	2	33	48	1 (3)*	2 (1)*	1 (1)	0 (1)*	2 (1)*	1 (1)	1 (1)
VVFDTSL	1	49	55	-1 (2)*	0 (1)*	0 (1)*	0 (0)*	0 (0)*	0 (0)	0 (0)*
DTSLQVKKAFF	2	52	62	N/A	1 (2)*	N/A	-3 (2)	0 (2)*	0 (2)*	0 (1)
QVKKAF	2	56	61	N/A	0 (1)*	N/A	-1 (1)	-1 (2)*	0 (1)*	N/A
QVKKAFF	2	56	62	0 (1)*	-1 (1)*	0 (1)	0 (1)	-1 (0)*	0 (0)	0 (0)*
LTITD	1	86	90	N/A	N/A	N/A	N/A	20 (3)	16 (4)	N/A
LTITDF	1	86	91	-9 (4)	4 (5)*	-13 (2)	-15 (2)	12 (2)	10 (2)	-3 (2)
FINILHRYYKSAL	2	91	103	N/A	N/A	N/A	N/A	-12 (4)	-4 (3)	N/A
FINILHRYYKSAL	3	91	103	N/A	-14 (2)	-7 (3)	-10 (2)	-13 (3)	-5 (3)	-7 (2)
INILHRYYKSAL	2	92	103	-1 (2)	-14 (2)	-8 (2)	-10 (2)	-14 (2)	-4 (3)	-7 (2)
INILHRYYKSAL	3	92	103	-2 (3)	-14 (2)	-8 (2)	-10 (2)	-14 (2)	-5 (3)	-7 (2)
VQIYE	1	104	108	3 (3)	12 (3)	8 (2)	9 (3)	8 (3)	5 (3)*	10 (2)
VQIYEL	1	104	109	N/A	6 (3)	5 (3)	5 (4)	2 (3)*	3 (3)	7 (2)
LEEHKIET	1	109	116	N/A	-13 (4)	-10 (2)	-11 (2)	-16 (3)	-6 (3)	-6 (1)
LEEHKIET	2	109	116	-7 (3)	-12 (4)	-10 (2)	-11 (2)	-16 (3)	-7 (3)	-6 (1)
LEEHKIETWRE	3	109	119	N/A	-13 (4)	N/A	N/A	-15 (4)	-7 (3)	N/A
WREVYLQD	1	117	124	N/A	-6 (4)	-4 (3)	-6 (3)	-2 (5)*	2 (3)	-7 (3)
WREVYLQD	2	117	124	-10 (3)	-7 (3)	-7 (2)	-7 (2)	-3 (2)*	1 (3)	-5 (2)
SFKPLVC	2	125	131	-6 (3)	0 (3)*	-3 (3)	-4 (4)	N/A	N/A	-2 (2)
FDAVSSL	1	139	145	0 (1)*	0 (1)*	0 (1)*	0 (1)*	N/A	0 (0)	0 (0)
YILTHKRILKF	2	165	175	-1 (1)	1 (0)*	-1 (0)	-1 (0)	N/A	1 (0)	0 (0)
YILTHKRILKF	3	165	175	-1 (1)*	0 (0)*	-1 (0)	-1 (0)	N/A	1 (0)	0 (0)
ILTHKRILKF	3	166	175	-1 (2)*	0 (1)*	-1 (1)	-1 (1)*	N/A	1 (1)*	0 (1)
FITEFPKPEF	1	179	188	-1 (4)*	2 (3)*	0 (3)	-2 (3)	N/A	5 (4)	-2 (3)
FITEFPKPEF	2	179	188	-1 (3)*	2 (3)*	-1 (3)	-2 (3)	N/A	4 (4)	-3 (3)
MSKSLEE	1	189	195	-4 (2)	N/A	-6 (1)	-6 (1)	N/A	-13 (4)	N/A
MSKSLEE	2	189	195	-2 (2)	N/A	-7 (1)	-7 (1)	N/A	-12 (3)	-23 (2)
LQIGT	1	196	200	-4 (2)	N/A	-8 (2)	-7 (1)	-27 (3)	-16 (3)	-26 (2)
LQIGTY	1	196	201	-8 (3)	N/A	-10 (1)	-8 (1)	-31 (4)	-15 (2)	-24 (3)
YANIAM	1	201	206	-7 (4)	-14 (4)	-9 (2)	-10 (2)	-19 (3)	-4 (3)	-10 (3)
ANIAM	1	202	206	N/A	-19 (3)	-12 (4)	-11 (3)	-22 (3)	N/A	-14 (3)
MVRTTTPVY	2	206	214	-3 (3)*	-14 (5)	-4 (3)	-5 (3)	-19 (3)	-8 (4)	-19 (3)
VRTTTPVY	1	207	214	-6 (3)	-17 (4)	-6 (2)	-6 (2)	-24 (3)	-10 (3)	-22 (3)
VRTTTPVY	2	207	214	-3 (3)	-17 (4)	-6 (2)	-6 (2)	-24 (3)	-10 (3)	-20 (2)
GIFVQHRVSAL	2	218	228	N/A	-25 (3)	N/A	N/A	N/A	-6 (3)	N/A
VQHRVSAL	1	221	228	-13 (4)	-34 (3)	-14 (2)	-16 (2)	-29 (3)	-6 (3)	-15 (3)
VQHRVSAL	2	221	228	-10 (4)	-33 (4)	-14 (3)	-16 (2)	-29 (3)	-6 (4)	-15 (3)
PVVDEKGRVVD	3	229	239	N/A	-16 (4)	-7 (2)	-9 (1)	-20 (3)	-12 (4)	N/A
PVVDEKGRVVDI	3	229	240	-9 (4)*	-17 (4)	-9 (1)	-9 (1)	-21 (3)	-12 (3)	-20 (2)
PVVDEKGRVVDIYSKF	2	229	244	-7 (2)	-17 (3)	-11 (1)	-11 (1)	-21 (2)	-11 (2)	-19 (2)
KGRVVDIYSKF	3	234	244	N/A	N/A	N/A	N/A	-20 (3)	N/A	-16 (2)
IYSKF	2	240	244	N/A	-18 (4)	N/A	N/A	-15 (3)	-9 (4)	N/A
DVINL	1	245	249	-2 (2)*	-16 (2)	-6 (1)	-7 (1)	-18 (1)	-7 (3)	-13 (1)
AAEKTYNNL	2	250	258	0 (4)*	-8 (3)	-6 (3)	-10 (3)	-19 (4)	0 (4)	-2 (4)
AAEKTYNNLD	2	250	259	N/A	-11 (5)	N/A	N/A	-22 (4)	-3 (4)	N/A
DVSVTKALQHRSHY	3	259	272	N/A	N/A	N/A	N/A	-24 (3)	N/A	N/A
DVSVTKALQHRSHYFEGGGKC	2	259	279	N/A	N/A	N/A	N/A	-20 (4)	N/A	-17 (3)
KCYLHETLET	2	278	287	N/A	-3 (2)*	-4 (2)	-3 (2)	N/A	-3 (2)*	N/A
YLHETL	1	280	285	-4 (3)	-5 (2)	-4 (2)	-5 (2)	N/A	-4 (2)	N/A
YLHETLET	2	280	287	-1 (1)	-3 (1)*	-2 (1)	-2 (1)	-12 (2)	-3 (1)	-14 (2)
HETLET	1	282	287	N/A	-3 (1)*	-3 (1)	-2 (1)	-10 (1)	-2 (1)	-11 (1)
ETIINRLVE	2	286	294	-2 (1)	0 (4)*	-1 (0)	-1 (0)	-2 (1)*	-1 (0)	-1 (1)
IINRLVE	2	288	294	-3 (1)	-2 (1)*	-2 (1)	-2 (1)	-2 (1)*	0 (1)	-1 (1)
IINRLVEA	2	288	295	N/A	-5 (2)*	-5 (1)	-5 (1)	4 (2)*	1 (1)	3 (2)
AEVHRLVV	2	295	302	-6 (3)	-9 (2)	-7 (1)	-9 (1)	2 (3)*	-1 (2)	1 (3)
AEVHRLVVVDEND	2	295	307	-4 (2)	-6 (2)	-5 (2)	-6 (1)	-1 (2)*	-3 (2)	-6 (1)
AEVHRLVVVDEND	3	295	307	N/A	-6 (2)	-5 (2)	-5 (1)	-1 (2)*	-2 (3)	-5 (2)
VDENDVVKGDVSL	2	303	315	N/A	N/A	N/A	N/A	N/A	-6 (2)	N/A
VDENDVVKGIVSL	2	303	315	-3 (2)	-8 (2)	-4 (1)	-3 (1)	-9 (2)	N/A	N/A
NDVVKG	1	306	311	N/A	-12 (5)	N/A	-7 (4)	-8 (3)	-9 (4)	-8 (3)
NDVVKGDVSL	2	306	315	N/A	N/A	N/A	N/A	N/A	-9 (2)	-19 (1)
DVVKGDVSL	2	307	315	N/A	N/A	N/A	N/A	N/A	-5 (3)	-13 (3)
VVKGIVSL	2	308	315	-3 (1)	-10 (2)	-5 (1)	-4 (1)	-12 (1)	N/A	N/A
VVKGDVSL	2	308	315	N/A	N/A	N/A	N/A	N/A	-9 (3)	-20 (2)
SDILQA	1	316	321	1 (1)*	-1 (0)*	0 (1)*	0 (0)*	-1 (0)*	-1 (0)	-2 (0)
QALVLTGGEKKP	2	320	331	N/A	2 (4)*	2 (3)	0 (3)	1 (4)*	4 (3)	-2 (3)
ALVLTGGEKKP	2	321	331	N/A	2 (5)*	N/A	N/A	2 (3)*	3 (4)	-1 (3)
LVLTGGEKKP	2	322	331	0 (4)*	2 (4)*	3 (4)	1 (3)	3 (3)*	4 (4)	-1 (4)
LVLTGGEKKP	3	322	331	1 (4)	3 (4)*	2 (4)	-1 (3)*	4 (3)*	4 (4)	0 (4)
