# Supplementary material for: Deconvoluting AMP-activated protein kinase (AMPK) adenine nucleotide binding and sensing
Source: J Biol Chem. 2017 Jun 14;292(30):12653–66. doi: 10.1074/jbc.M117.793018 (PMC5535039; doi:10.1074/jbc.M117.793018)
Supplement: Supplemental Data [file 10.1074_M117.793018_jbc.M117.793018-3.pdf]

Supplemental Table 3: ΔHDX-MS peptide list for the α1- and β2-subunits. Resolved peptides are listed in the first column and % changes in HDX are indicated by numbers and heat map color.

Wild Type α1

				2.5mM AMP	2.5mM ATP	4.5mM ATP 0.4mM ADP 0.04mM AMP (Non-stress AXP mixture)	3.8mM ATP 1.0mM ADP 0.3mM AMP (Energy stress AXP mixture
Sequence	Charge	Start	End	WT AMP alpha1	WT ATP alpha1	WT Mix1 alpha1	WT Mix2 alpha1
FRHPHIIKL	2	88	96	-5 (1)	-6 (2)	-6 (1)	-6 (1)
FRHPHIIKL	3	88	96	-6 (1)	-6 (1)	-6 (1)	-6 (1)
YQVISTPSD	1	97	105	7 (3)	2 (4)*	4 (2)	3 (2)
YQVISTPSDIF	1	97	107	5 (3)	1 (3)	2 (2)	2 (2)
YQVISTPSDIF	2	97	107	5 (3)	1 (3)	3 (2)	2 (2)
YVSGGEL	1	112	118	-9 (2)	-11 (3)	-10 (2)	-11 (3)
FDYICKNGRL	2	119	128	-7 (3)	-10 (4)	-9 (2)	-10 (3)
FDYICKNGRL	3	119	128	-9 (4)	-7 (4)	-10 (2)	N/A
DAHMNAKIADFGLSNM	2	165	180	-12 (3)	-28 (4)	-19 (2)	-18 (4)
DAHMNAKIADFGLSNM	3	165	180	-13 (3)	-24 (4)	-18 (2)	-18 (3)
NAKIADFGLSNM	1	169	180	-7 (4)	-25 (5)	-15 (4)	-16 (4)
NAKIADFGLSNM	2	169	180	-7 (3)	-23 (5)	-16 (3)	-16 (3)
FGLSNM	1	175	180	1 (4)*	-25 (5)	N/A	-11 (4)
LRTSCGSPNYAAPEVISGRL	3	187	206	-4 (4)	-15 (5)	-12 (3)	-11 (4)
YAGPEVDIWSSG	1	207	218	-8 (3)	-13 (3)	-11 (1)	-11 (2)
IWSSGVIL	1	214	221	N/A	-10 (3)	-8 (1)	-8 (1)
ALLCGTLPFDDDHVPTL	2	223	239	0 (2)	N/A	-2 (2)	-2 (2)
LCGTLPFDDDHVPTL	2	225	239	2 (3)	N/A	0 (2)	0 (2)
CGTLPFDDDHVPTL	2	226	239	3 (3)	-1 (3)	0 (3)	-1 (2)
FKKICDGIF	1	240	248	10 (4)	8 (3)	10 (3)	8 (3)
FKKICDGIF	3	240	248	10 (3)	8 (4)	N/A	7 (3)
IREHEWFKQDLPKYLF	4	279	294	N/A	-6 (3)	-5 (2)	-5 (3)
IREHEWFKQDLPKYLFPEDPSY	4	279	300	1 (4)	N/A	-1 (3)	-2 (3)
FKQDLPKYLFPEDPSYSSTM	3	285	304	9 (4)	N/A	N/A	5 (3)
IDDEALKEVCE	2	305	315	-1 (1)	N/A	-2 (1)	-2 (1)
YNRNHQDPLAVAY	2	329	341	2 (3)	-2 (3)	0 (2)	0 (2)
YNRNHQDPLAVAY	3	329	341	2 (3)*	-1 (3)*	0 (2)	0 (2)
YNRNHQDPLAVAYHLIIDNRRIMNE	4	329	353	N/A	-3 (3)	N/A	-1 (2)
YNRNHQDPLAVAYHLIIDNRRIMNEAKD	4	329	356	2 (3)*	-2 (5)*	-1 (2)	-2 (2)
HLIIDN	1	342	347	N/A	N/A	N/A	-4 (2)
FYLATSPPDSF	1	357	367	12 (5)	4 (4)	7 (5)	4 (3)
FYLATSPPDSF	2	357	367	8 (5)	N/A	N/A	5 (3)
FYLATSPPDSFL	2	357	368	9 (5)	N/A	5 (5)	4 (3)
YLATSPPDSF	1	358	367	11 (5)	N/A	7 (5)	N/A
YLATSPPDSFL	1	358	368	10 (5)	4 (5)*	6 (5)	4 (4)*
AEVCRAIKQLD	2	428	438	-1 (2)*	N/A	N/A	0 (3)
AEVCRAIKQLDYE	2	428	440	0 (0)*	N/A	N/A	0 (1)*
IKQLDYE	2	434	440	0 (1)*	N/A	N/A	0 (2)
WKVVNPYYL	2	441	449	1 (2)*	N/A	N/A	1 (2)*
YSKMSLQL	1	461	468	1 (2)*	0 (3)*	1 (2)*	0 (2)*
YSKMSLQL	2	461	468	0 (1)	-1 (2)*	0 (1)*	0 (0)*
YQVDSRT	2	469	475	3 (4)	3 (3)*	1 (3)*	1 (3)*
YQVDSRTYL	2	469	477	2 (2)	1 (2)*	1 (2)*	1 (2)*
DSSPVDLTPRPGSHTIEF	2	535	552	6 (3)	4 (2)	4 (1)	4 (2)
FEMCANL	1	553	559	0 (1)*	-1 (2)*	0 (1)*	0 (1)*
IKILAQ	2	560	565	0 (1)*	1 (1)*	0 (1)	0 (0)*

Wild Type β2

				2.5mM AMP	2.5mM ATP	(mix 1) 4.5mM ATP 0.4mM ADP 0.04mM AMP	(mix 2) 3.8mM ATP 1.0mM ADP 0.3mM AMP
Sequence	Charge	Start	End	WT AMP beta2	WT ATP beta2	WT Mix1 beta2	WT Mix2 beta2
FSLPDSKLPGDKE	3	46	58	6 (5)*	3 (5)*	4 (5)	4 (4)*
FSLPDSKLPGDKEF	3	46	59	N/A	N/A	6 (4)	N/A
FVSWQQDLED	1	59	68	9 (5)*	4 (5)*	6 (4)	5 (5)
VFISGSFNNW	1	90	99	N/A	-8 (4)	-2 (4)*	-6 (3)
ISGSFNNW	1	92	99	-12 (5)	-7 (3)	-7 (3)	-9 (3)
NNWSTKIPLIKSHNDF	3	97	112	-5 (4)	N/A	-5 (3)	N/A
STKIPLIKSHNDF	2	100	112	-3 (4)	-4 (3)	-2 (2)	-2 (2)
STKIPLIKSHNDF	3	100	112	-3 (3)	-4 (4)	-2 (2)	-2 (2)
STKIPLIKSHNDF	4	100	112	-3 (4)*	N/A	-1 (3)	2 (3)
VAILDLPEGEHQYKF	2	113	127	-3 (3)	-3 (3)	-3 (2)	-3 (2)
VAILDLPEGEHQYKF	3	113	127	-3 (2)	-4 (3)	-3 (2)	-3 (2)
LDLPEGEHQYKF	2	116	127	-6 (4)	-7 (4)	-7 (3)	-6 (3)
LDLPEGEHQYKF	3	116	127	-7 (4)	-4 (5)	-5 (3)	-7 (3)
FVDGQWVHDPSEPVVT	2	128	143	-10 (5)	-8 (4)	-8 (2)	-9 (3)
WVHDPSEPVVT	2	133	143	-10 (5)	N/A	N/A	N/A
WVHDPSEPVVTSQL	2	133	146	-6 (4)	-6 (4)	-5 (4)	-6 (4)
GTINNLIHVKKSDF	2	147	160	-4 (3)	-5 (4)*	-4 (4)	-5 (4)
GTINNLIHVKKSDF	3	147	160	-4 (3)	-4 (4)*	-4 (4)*	-6 (3)
LIHVKKSDF	2	152	160	2 (4)	0 (4)*	0 (4)*	-1 (3)*
IHVKKSDF	2	153	160	4 (3)	1 (4)*	3 (4)*	1 (3)*
DALKLDSM	1	164	171	9 (5)	N/A	7 (4)	5 (4)*
FRSEERFKSPPILPPHL	3	196	212	5 (3)	N/A	N/A	2 (3)*
VILNKDTNISCD	2	215	226	N/A	N/A	N/A	2 (4)*
PALLPEPNHVMLNHL	2	227	241	3 (3)	0 (4)*	2 (2)	1 (2)*
PALLPEPNHVMLNHL	3	227	241	3 (3)	2 (3)*	2 (2)	1 (2)*
YALSIKDSVM	2	242	251	8 (3)	2 (3)*	3 (3)	2 (3)*
YALSIKDSVMVL	2	242	253	6 (3)	1 (3)*	2 (2)*	2 (2)*
SATHRYKKKYVTTL	2	254	267	1 (1)	N/A	0 (1)*	0 (0)*
SATHRYKKKYVTTL	3	254	267	1 (1)	0 (2)*	0 (1)*	0 (0)*
SATHRYKKKYVTTLL	2	254	268	1 (1)	N/A	N/A	0 (1)*
SATHRYKKKYVTTLL	3	254	268	1 (1)	0 (2)*	0 (0)*	0 (0)
LYKPI	1	268	272	0 (4)*	N/A	N/A	-1 (3)*
LYKPI	2	268	272	0 (2)*	1 (2)*	-2 (3)*	-1 (3)*
